# The Effects of a Mindfulness-Based Intervention on Mental Health Outcomes in Pregnant Woman: A Systematic Review and Meta-Analysis

**DOI:** 10.1097/jnr.0000000000000586

**Published:** 2023-11-28

**Authors:** Yanti Puspita SARI, Yu-Yun HSU, Tram Thi Bich NGUYEN

**Affiliations:** 1 MSN, RN, Doctoral Candidate, Department of Nursing, College of Medicine, National Cheng Kung University, Taiwan; and Assistant Professor, Maternity and Child Health Nursing Department, Faculty of Nursing, Universitas Andalas, Padang, West Sumatra, Indonesia; 2 PhD, RN, Professor, Department of Nursing, College of Medicine, National Cheng Kung University, Taiwan; 3 MS, RN, Doctoral Student, Department of Nursing, College of Medicine, National Cheng Kung University, Taiwan; and Lecturer, Medical Simulation Center, Duy Tan University, Vietnam.

**Keywords:** mindfulness-based intervention, pregnant women, stress, anxiety, depression

## Abstract

**Background:**

Pregnancy stress, anxiety, and depression increase the risk of short-term and long-term health problems for the mother and fetus. Mindfulness-based intervention (MBI) is one of the most popular, nonpharmacological interventions used to treat mental health problems. The results of prior research indicate MBI has a less consistent effect on mental health problems in pregnant women.

**Purpose:**

The purpose of this systematic review and meta-analysis was to clarify and determine the effect of MBI on mental health outcomes in pregnant women.

**Methods:**

Six databases, including Embase, Ovid MEDLINE, CINAHL, EBSCOhost, Cochrane Library, and ScienceDirect, were searched from their dates of inception to November 2021. Google Scholar was also used for the literature inquiry. The inclusion criteria followed the PICO (Patient/Problem, Intervention, Comparison, and Outcome) model in terms of only including studies that used mindfulness therapy, reported mental health outcomes, and applied randomized controlled trial and quasi-experimental approaches. The Cochrane risk of bias tool was applied to evaluate the quality of the studies. Review Manager 5 software with random effect with a standardized mean difference (SMD) was used to analyze level of effect.

**Results:**

Thirteen studies (10 randomized controlled trials and three quasi-experimental studies) were included. MBI was found to have a small effect on mental health outcomes in pregnant women (*p* < .0001, SMDs = −0.48, 95% CI [−0.74, −0.22], *I*^2^ = 87%). Specifically, MBI had moderate effects on stress and anxiety (SMDs = −0.59, 95% CI [−1.09, −0.09], and SMDs = −0.55, 95% CI [−1.00, −0.10], respectively) and no significant effect on depression (SMDs = −0.33, 95% CI [−0.74, 0.08]).

**Conclusions:**

MBIs have a small but notable effect on mental health in pregnant women. The high heterogeneity found in this review may reflect the different types and durations of interventions used. Notably, none of the studies in the review examined intervention effects by trimester. Future research should use larger sample sizes and assess the effects of therapy for each trimester of pregnancy.

## Introduction

Instability in mental health during pregnancy has become a health issue. Previous studies have found pregnant women experience a high incidence of mental health problems such as stress, anxiety, and depression and that these problems impact the postpartum period (Pampaka et al., 2018; Spry et al., 2020). It is estimated that the prevalence of anxiety and depression during each trimester of pregnancy ranges from 18.2% to 24.6% and 7.4% to 12.8%, respectively (Dennis et al., 2017).

Stress, anxiety, and depression during pregnancy may increase risks to the well-being of both mothers and their fetuses (Li et al., 2016). For example, pregnant women with mental health problems have been found to be at a higher risk of giving birth prematurely (Spry et al., 2020; Uguz et al., 2019). In addition, their children have been found to face a higher risk of experiencing mood disorders (Goodman, 2019; Savory et al., 2020). In addition, stress and anxiety during pregnancy increase the risk of postpartum depression in mothers (Nath et al., 2019; Verreault et al., 2014).

Providing effective and efficient interventions to reduce stress, anxiety, and depression in pregnant women is crucial (Goodman, 2019). Using pharmaceutical therapy for negative emotions is usually not recommended because of the possible side effects of the drugs on the health of both mothers and their fetuses (Galbally et al., 2020; Hardy & Reichenbacker, 2019). Therefore, nonpharmacological interventions have been recommended for pregnant women with negative emotions (Trinh et al., 2022). Mindfulness-based intervention (MBI) has been one of the most popular nonpharmacological intervention options in recent years.

MBI was first developed in the 1970s (Shapero et al., 2018). Generally, the target populations of this intervention have been children with special needs, obese patients, and mothers during the perinatal period (Noetel et al., 2017). The goal of MBIs is to increase awareness of one's body, mind, and feelings to create a sense of calm, and it has been widely used to reduce stress (Gamaiunova et al., 2022), anxiety, and depression (Gomutbutra et al., 2022). The two “gold standards” for MBIs, namely, mindfulness-based cognitive therapy (MBCT) and mindfulness-based stress reduction (MBSR), have been widely used, including with perinatal mothers (Shapero et al., 2018). Many investigators have recently found MBIs can decrease symptoms of stress, anxiety, and depression significantly in pregnant mothers (Shi & MacBeth, 2017).

However, one previous systematic review and meta-analysis study by Lever Taylor et al. (2016) reported less consistent effects of MBI on stress, anxiety, and depression, with significant yet small effects on stress and anxiety and a moderate effect on depression in perinatal mothers. In addition, the design of the individual studies included in that study varied greatly (Lever Taylor et al., 2016). Furthermore, another systematic review did not perform a meta-analysis but rather focused on narrative analyses related to the findings of each study because of the wider variety of study designs included in the review (Shi & MacBeth, 2017).

On the basis of the findings of the abovementioned systematic reviews, the evidence regarding the effects of MBI on stress, anxiety, and depression reduction in pregnant women is inconclusive. Hence, additional systematic reviews and meta-analyses are needed. Therefore, this review was developed to investigate, in greater detail, the type, duration, and timing of administration of MBI in pregnant women.

### Study Aims

This systematic review and meta-analysis was designed to determine the effect of MBIs on mental health outcomes during pregnancy in terms of three indicators: stress, anxiety, and depression. The research question to be answered is “Did the mindfulness-based intervention (I) affect mental health outcomes (e.g., stress, anxiety, and depression; O) in pregnant women (P)?”

## Methods

### Search Strategy

Six electronic databases, including Embase, Ovid MEDLINE, CINAHL, EBSCOhost, Cochrane Library, and ScienceDirect, were searched from their inception through November 2021. The search engine Google Scholar was applied to sort articles; keywords and vocabulary control from Emtree, MeSH, and CINAHL subject headings were used; and the keywords used were as follows: pregnant women or perinatal women (P), mindfulness (I), and mental health, stress, anxiety, or depression (O).

### Inclusion Criteria

The inclusion criteria for studies analyzed in this study included the following: (a) used mindfulness as intervention and therapy as usual, Lamaze intervention, or psychological therapy as the comparator; (b) reported mental health outcomes (e.g., stress, anxiety, and depression) during pregnancy; (c) involved female participants free of physical and psychological complications carrying a singleton pregnancy; (d) published in English in peer-reviewed journals; and (e) used a randomized controlled trial (RCT) or quasi-experimental design.

### Study Selection

The bibliographic management software package EndNote X9 was used to organize the bibliographies and references and check for duplications. Two of the authors performed the screening process independently. First, the title and abstract were screened to identify potentially eligible studies. Second, full-text articles were screened to determine eligibility based on the inclusion criteria. The third author was consulted to resolve any disagreement between the two reviewers.

### Data Extraction

The Joanna Briggs Institute appraisal tool was used to judge the trustworthiness of research evidence in the included studies, with the critical appraisal process performed independently by two of the authors. One author extracted the data independently, and a second author checked for accuracy. Any disagreement was resolved through discussion and, if necessary, through consultation with experts (Table 2). Information extracted from the studies included author names, setting, number of participants, screening tool(s) used, outcomes, comparison of interventions and control therapies, follow-up time(s), and results.

### Quality Assessment

The Cochrane risk bias tool was used to evaluate risk of bias in the included studies. This tool addresses the eight facets of random sequence generation, allocation concealment, blinding of participants and personnel, blinding of outcome assessment (self-reported outcome), blinding of outcome assessment reaction time, incomplete outcome data, selective reporting, and other biases. Two of the authors separately followed the instructions in the Cochrane Collaboration tool to assess risk of bias based on the following six domains and seven items: (a) the sequence allocation used for the intervention and control groups (selection bias), (b) the random sequence method used to determine the intervention and control groups (selection bias), (c) blinding of participants and personnel (performance bias), (d) blinding of outcome assessment (detection bias), (e) incomplete outcome data (attrition bias), (f) selective reporting (reporting bias), and (g) other sources of bias (other bias). Bias was assessed as high, low, or unclear for each of the elements from the six domains.

### Statistical Analysis

Meta-analyses were conducted using Review Manager (computer program) Version 5.4 software. The purpose of this meta-analysis was to determine differences in the effect of MBIs on mindfulness compared with other treatments in pregnant women. Standardized mean differences (SMDs) with 95% confidence intervals (CIs) were calculated, as the tools were used to measure relative differences in outcomes. SDM effect size was classified as follows: 0.2–0.5 = small effect, 0.5–0.8 = moderate effect, and > 0.8 = large effect. When heterogeneity values exceeded 50%, a random-effects model was applied (Higgins et al., 2019).

## Results

### Study Selection

**Figure 1.**
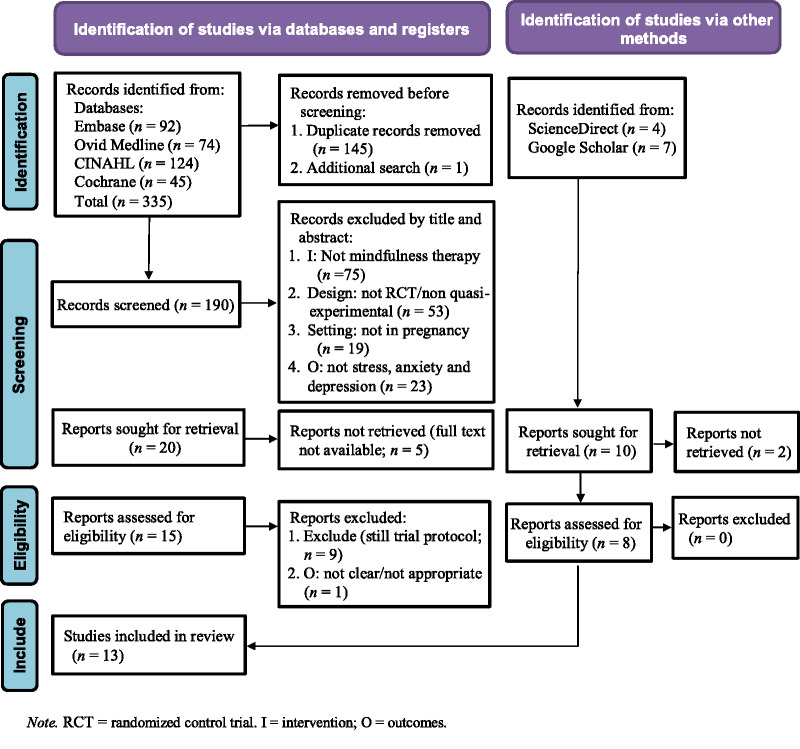
Flowchart of Preferred Reporting Items for Systematic Reviews and Meta-Analyses

The initial database search identified 335 studies, and a subsequent manual search identified an additional 11 studies. Two hundred studies (190 from the databases + 10 from the manual search) remained after deleting 146 duplicates. After screening the titles and abstracts of these articles, 30 were designated for full-text retrieval. Five of the articles could not be retrieved, leaving 23 full-text articles available for eligibility assessment. Thirteen studies were subsequently qualified and included in the review (Figure 1). Ten of the included studies were RCT, and three were quasi-experimental.

### Data Extraction

The results of the review of the research characteristics of the included studies are shown in Table 1. The included studies covered 1,032 pregnant women (*n* = 518 in the intervention groups, *n* = 514 in the control groups). Most of the studies were conducted in the United States (*n* = 5), with the remainder (*n* = 8) conducted in Asia (China: *n* = 1, Taiwan: *n* = 2, Iran: *n* = 2, and India *n* = 1), Australia (*n* = 1), and Sweden (*n* = 1). Mental health outcomes consisted of three indicators: stress, anxiety, and depression.

**Table 1.** Summary of the Characteristics of Included Studies

No.	Author (Year)	Country	Design	Sample Size		Therapy	Time to Follow-Up	Outcomes	Instruments	Experimental		Control	
				Experiment	Control					*M*	*SD*	*M*	*SD*
1.	Beattie et al. (2017)	Australia	RCT	24	24	Mindfulness in Pregnancy Program for eight sessions, more than 2 hours for each session versus Pregnancy Support Program	Six weeks after finishing the intervention	Stress	Perceived Stress Scale (PSS)	−3.40	6.48	−4.20	9.11
								Depression	Edinburgh Postnatal Depression Scale (EPDS)	−5.30	7.14	−4.20	9.11
2.	Dimidjian et al. (2016)	Colorado, USA	RCT	43	43	Mindfulness-based cognitive therapy–perinatal depression for eight sessions, 2 hours each session versus TAU		Depression	EPDS	−1.31	5.92	1.32	6.63
3.	Epel et al. (2019)	United States	A quasi-experimental trial	89	82	Mindful Moms Training for eight sessions, 2 hours each session versus TAU		Stress	PSS	−3.50	9.10	−1.40	10.70
								Anxiety	Pregnancy-Related Anxiety Scale (PRAS)	−0.10	0.90	−0.10	0.95
4.	Guardino et al. (2014)	United States	RCT	21	20	Mindful awareness practices for 6 weeks series, 2 hours each series versus TAU	Six weeks after finishing the intervention	Stress	PSS	−4.51	8.38	−4.11	12.28
								Anxiety	PRAS	−3.98	3.21	−1.75	4.39
5.	Lonnberg et al. (2020)	Sweden	RCT	96	97	Mindfulness-Based Childbirth and Parenting (MBCP) for nine sessions, 2 hours and 15 minutes each session versus Lamaze intervention		Stress	PSS	−6.07	10.13	−3.80	11.61
								Depression	EPDS	−3.61	6.52	1.77	8.03
6.	Muthukrishnan et al. (2016)	Indian	Quasi-experimental	37	37	Mindfulness meditation on perceived stress for 10 sessions, 30 minutes each session		Stress	PSS	−11.54	2.35	1.52	3.45
7.	Pan, Chang, et al. (2019)	Taiwan	RCT	35	39	MBCP for eight sessions, 30 minutes each session versus standard presentation on physiological and psychological information practice skills		Stress	PSS	−3.77	9.00	0.49	8.24
								Depression	EPDS	−2.98	6.49	0.03	5.64
8.	Pan, Gau, et al. (2019)	Taiwan	RCT	51	45	MBCP for nine sessions, 3 hours each session versus conventional childbirth education		Stress	PSS	15.61	6.24	13.69	5.76
								Depression	EPDS	9.75	0.59	9.02	0.68
9.	Vieten & Astin (2008)	California, USA	RCT	13	18	Mindfulness-based stress reduction (MBSR) for eight sessions, 2 hours each session versus TAU		Stress	PSS	−4.20	8.25	−0.20	7.10
								Anxiety	State–Trait Anxiety Inventory (STAI)	−8.40	15.30	0.00	13.85
10.	Zarenejad et al. (2020)	Iran	RCT	30	30	MBSR for six sessions, two times per weeks, 60 minutes versus no specific intervention		Depression	EPDS	−4.20	11.50	3.00	10.10
	Anxiety	Pregnancy-Related Anxiety Questionnaire	−28.40	98.90	−7.20	62.95
11.	Zemestani & Fazeli Nikoo (2020)	Iran	RCT	19	19	Mindfulness-based cognitive therapy for eight sessions, 2 hours each session versus no interventions	1-month follow-up after finishing the intervention	Anxiety	Beck Anxiety Inventory	−12.77	6.22	0.07	7.27
								Depression	Beck Depression Inventory-II	−20.32	7.72	2.20	9.63
12.	H. Zhang & Emory (2014)	African American population, no specific country	RCT	34	30	Mindful Motherhood intervention for eight sessions, 1 hour each session versus TAU		Stress	PSS	−4.20	12.56	−0.60	12.73
								Depression	Beck Depression Inventory-II	−1.60	15.80	1.00	12.19
13.	J.-Y. Zhang et al. (2019)	Chinese	Quasi-experimental	26	30	MBSR for eight sessions, 2 hours each session versus TAU		Stress	Pregnancy Stress Rating Scale	−1.98	5.46	3.44	6.84
								Anxiety	The Chinese version of STAI	−2.38	6.74	0.81	6.92
								Depression	Self-Rating Depression Scale	−0.19	11.36	−0.09	9.87

*Note.* RCT = randomized controlled trial; TAU = therapy as usual.

Various instruments were used to measure stress, anxiety, and depression. For stress, the Perceived Stress Scale was used in nine studies, and one study used the Pregnancy Stress Rating Scale. For anxiety, one study used the Pregnancy-Related Anxiety Questionnaire, two used the State–Trait Anxiety Inventory, one used the Chinese version of the State–Trait Anxiety Inventory, two used the Pregnancy-Related Anxiety Scale, and one used the Beck Anxiety Inventory scale. For depression, two studies used the Beck Depression Inventory-II, one used the Self-Rating Depression Scale, and six used the Edinburgh Postnatal Depression Scale.

As shown in Table 1, the intervention groups in the 13 studies received various MBIs, with three studies having used MBSR (Vieten & Astin, 2008; Zarenejad et al., 2020; J.-Y. Zhang et al., 2019). The MBSR interventions focused on emotional regulation, breathing, sitting and walking meditation, yoga movements, and body scans. The MBSR methods used in these studies were modified to reflect a focus on managing the fear of childbirth and pregnancy complications (Zarenejad et al., 2020).

Two studies used MBCT interventions (Dimidjian et al., 2016; Zemestani & Fazeli Nikoo, 2020). In both studies, the MBCT interventions focused on the awareness regulation of emotions and body sensations specifically in the context of pregnant women. In addition, love meditation was introduced to increase the awareness of the relationship between the mother and the fetus that included psychoeducation on the possibility of overcoming difficulties experienced during childbirth (Dimidjian et al., 2016).

Five of the studies employed MBIs specifically related to parenting, awareness in pregnancy, and motherhood. These included the Mindfulness-Based Childbirth and Parenting Program (Lonnberg et al., 2020; Pan, Chang, et al., 2019; Pan, Gau, et al., 2019), the Mindfulness in Pregnancy Program intervention (Beattie et al., 2017), and the Mindful Moms Training therapy (Epel et al., 2019). Body scan meditation is used in all three along with therapy sessions focused on increasing awareness of the baby and dealing with labor pains. The Mindfulness in Pregnancy Program intervention emphasizes expanding the ability to recognize body characteristics, regulating emotions, meditation, body scanning, and increasing awareness of the baby. Mindful Moms Training therapy focuses on stress reduction; awareness of breathing, body, and emotions; and awareness of nutritional needs.

The number of MBI sessions used in the studies ranged from six to 10, with most (*n* = 10) using eight. Two studies used six sessions (Guardino et al., 2014; Zarenejad et al., 2020), and one used 10 sessions (Muthukrishnan et al., 2016). The length of each session of therapy ranged from 30 minutes to 2 hours. The initial session of the MBI took place at about 20 gestational weeks. Several studies also carried out post-follow-up measurements in the postpartum period (Dimidjian et al., 2016; Lonnberg et al., 2020; Pan, Chang, et al., 2019; Vieten & Astin, 2008).

### Report of Bias Assessment

**Figure 2.**
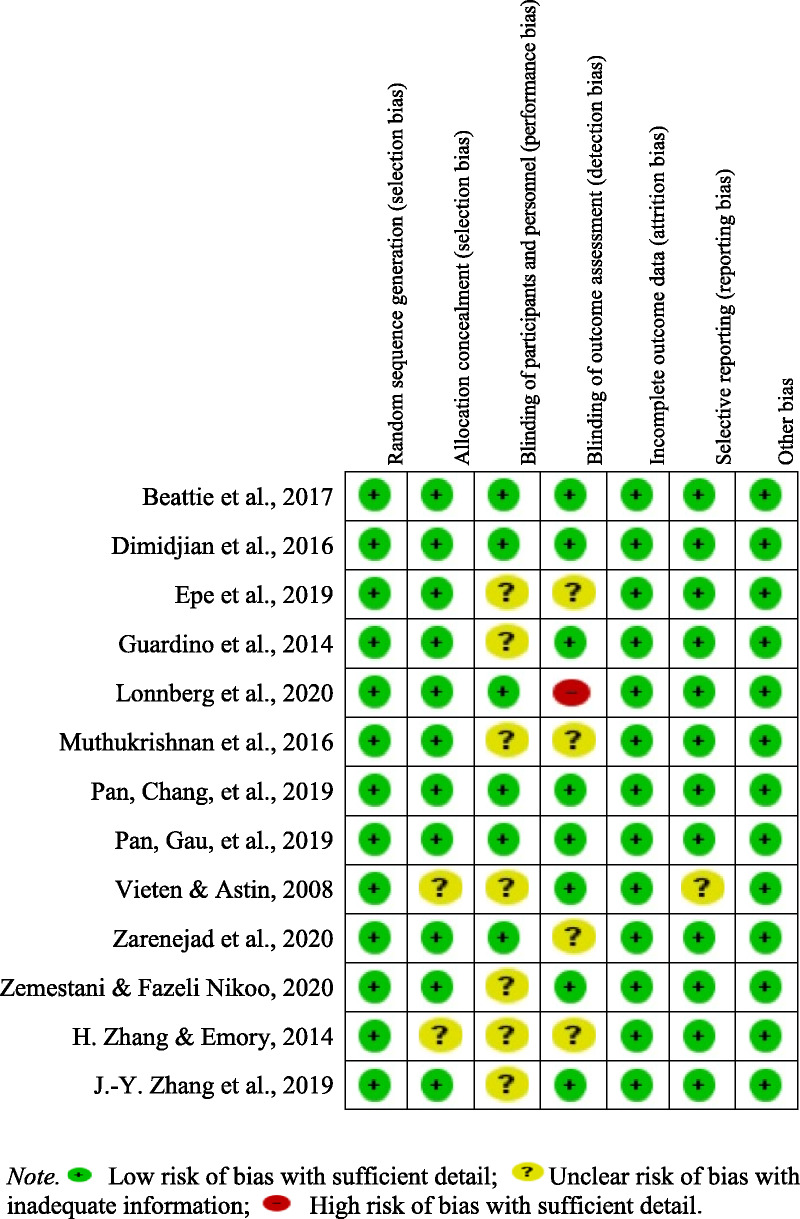
Risk of Bias in Each Included Study

The risk of bias results, indicating low risk of selection bias in most studies, are shown in Figure 2. The study participants were randomly assigned using a computerized system into either the experimental or comparison group. In addition, nearly all of the studies reported single-blind measures of therapeutic outcomes. All of the studies identified an attrition rate ranging from 6% to 27%.

**Figure 3.**
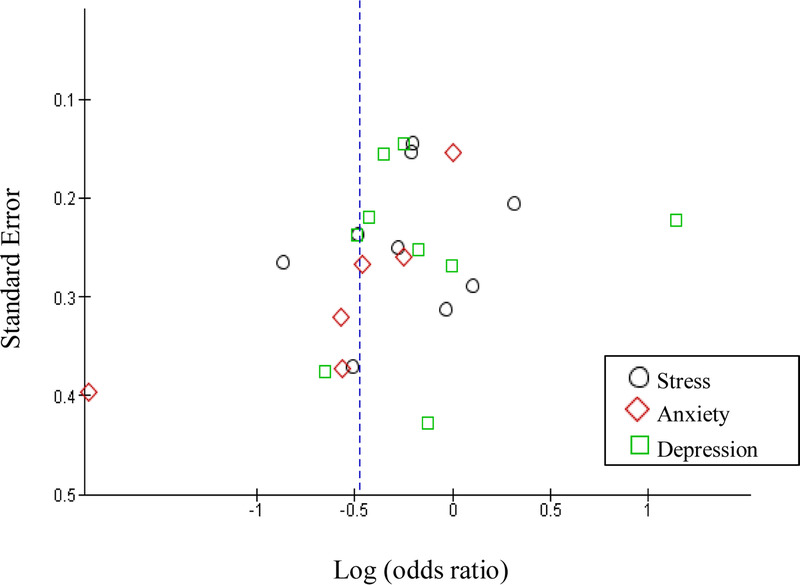
Funnel Plot for Publication Bias

The funnel plot (Figure 3) based on the overall effect of MBI in reducing mental health outcomes using SMD presented an asymmetric plot, indicating the studies may be impacted by publication bias and revealing a significant difference in effect size among the studies as well as overly small sample sizes (Higgins et al., 2019).

### Outcomes: Effects of Intervention

This meta-analysis used a random-effects model to address the high heterogeneity (*I*^2^ = 87%). On the basis of a forest plot (Table 2), the MBIs had a small-to-medium effect on mental health outcomes (SMDs = −0.48, 95% CI [−0.74, −0.22], *I*^2^ = 87%), indicating the MBIs significantly reduced mental health problems in the participants.

**Table 2.**
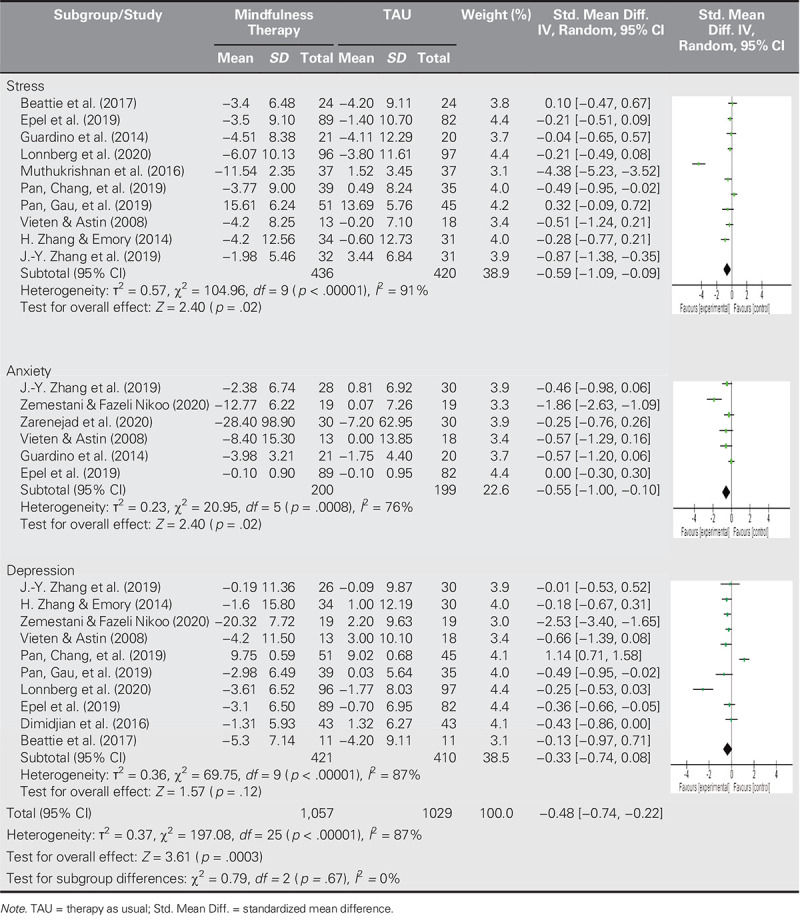
Forest Plot

Subgroup analyses of the mental health outcomes for stress, anxiety, and depression were also conducted (Table 2). The 10 studies included in the analysis of the effects of MBI on stress reflected a moderate effect of MBI on stress (SMDs = −0.59, 95% CI [−1.09, −0.09], *p* = .02, heterogeneity *I*^2^ = 91%). Similarly, the six studies included in the analysis of the effects of MBI on anxiety reflected a moderate effect of MBI on anxiety (SMDs = −0.55, 95% CI [−1.00, −0.10], *p* = .02, heterogeneity *I*^2^ = 76%). However, the 10 studies included in the analysis of the effects of MBI on depression reflected no significant effect (SMDs = −0.33, 95% CI [−0.74, 0.88], *p* = .12, *I*^2^ = 87%).

## Discussion

This review and meta-analysis included 10 studies that employed an RCT design and three that employed a quasi-experimental design. The reviews identified a significant but small effect of MBI on mental health outcomes, which is similar to the previous systematic review and meta-analysis. This finding contrasts with the findings of Lever Taylor et al. (2016), who identified a small effect of MBI on stress and anxiety and a moderate effect on depression.

The results of several MBI clinical studies designed to measure brain activity complement the findings of our review. Stress is experienced by most people, representing a natural response and reaction to environmental stimuli (Gamaiunova et al., 2022). One clinical study states that mindfulness interventions effectively reduce stress by improving brain function and balance, attention, and memory. MBI influenced the activity of the hypothalamic–pituitary–adrenocortical system, which reduced cortisol secretion levels (Aguilar-Raab et al., 2021) and subsequently improved emotional control and sleep quality in individuals who followed an 8-week MBSR intervention (Felsch & Kuypers, 2022). MBI affects functions in the anterior cingulate cortex, ventromedial prefrontal cortex, hippocampus, amygdala, and other sections of the brain (Gamaiunova et al., 2022; Nakamura et al., 2021). On the other hand, mindfulness may increase other psychological effects such as self-care capacity, autonomy, and body awareness (Macaulay et al., 2022).

Similar to its effect on stress, MBI was shown to have a moderate impact on anxiety in the meta-analysis. This result aligns with the most recent study by Lever Taylor et al. (2016), which identified a significant effect of an MBI on anxiety in pregnant mothers.

Unlike the outcomes for stress and anxiety, MBI was found to have no significant effect on depression in this study. This contrasts with Lever Taylor et al. (2016), who found a moderate effect of MBI on depression. The difference in findings may be attributable to several factors. First, the timing of depression measurements may differ significantly between the two studies. For example, Dadi et al. (2020) found the prevalence of antenatal depression ranged from 4.7% to 8.1% in the second and third trimesters of pregnancy (Dadi et al., 2020), with trimester shown to be an important factor influencing depression. Second, differences in the instruments used to measure depression may have affected the measurement results (Bretschneider et al., 2018). Third, a previous study found trainer ability to train participants to be an important factor influencing the success of mindfulness therapy (Dimidjian & Segal, 2015). Finally, although a recent clinical study showed a positive effect of MBI on depression through the mechanism of decreasing activation of the posterior cingulate cortex of the brain, the small number of samples (*n* = 16) and the absence of controls mean further research is needed to confirm these results (Williams et al., 2020).

As discussed above, differences exist among the included studies in terms of the MBI effects on stress, anxiety, and depression. We found a significant impact of MBI on maternal mental health symptoms, especially stress and anxiety, but no impact on depression. Notably, more recent research employed interventions that were more comprehensive involving techniques for calming the mind and regulating breathing, improving communication skills, and facing fear of labor pain. In addition, average therapy duration in this review study differs significantly from that reported in previous meta-analyses. For example, Lever Taylor et al. (2016) reported an average duration of 30–40 minutes per session, whereas this review found a range of 60–120 minutes.

This meta-analysis found high variation (*I*^2^ > 50%) among MBIs and instrument types. The four types of MBIs used included: MBIs focused on parenting, awareness in pregnancy and motherhood, MBSR interventions, MBCT interventions, and mindfulness awareness interventions.

Despite the variety of MBIs in this review, similarities among each were identified. The MBIs all involved a series of treatments focused on awareness, mind, and body that are aimed to relax the psychological condition of pregnant women. Forms of mindfulness therapy include mindful movement, sitting and walking meditations, loving-kindness meditations, stress reduction focused on acceptance-based coping, breathing, body, thoughts, and emotions.

### Strengths

This review and meta-analysis involved 10 studies that employed an RCT design and three that employed a quasi-experimental design. We considered studies not previously included in meta-analyses and conducted analyses that were more advanced than used in previous studies. Thus, the results of this meta-analysis offer considerable new evidence in support of the effectiveness of MBIs on mental health outcomes in pregnant mothers.

### Limitations

This review was affected by several limitations. First, although MBIs were found to significantly improve mental health in pregnant women, especially in terms of stress and anxiety, potential differences in effect by trimester were not considered. Second, participants in the included studies were not distinguished between primiparous and multiparous individuals. Third, this review did not differentiate the effects of MBIs by age category (e.g., young mothers, advanced-age mothers). Fourth, wide variations in the measurement tools used to examine mental health in the included studies may contribute to the high heterogeneity observed in this review. Finally, the review did not include qualitative studies, and thus, the results do not explain the meaning, experience, or perspective of participants.

### Future Research

Further research is necessary to identify differences in MBI effect based on age group, parity, and previous pregnancy history. In addition, the effects of MBIs during each trimester of pregnancy should be explored. In addition, the cultural issues/considerations related to MBIs have not been adequately addressed in the literature. On the basis of the authors' understanding, MBIs were originally inspired by Hinduism/Buddhism meditation practices. Thus, despite their good effects in practice, the ability to implement MBIs may be influenced by cultural concerns and beliefs.

### Clinical Implications

MBIs offer a therapeutic option for reducing mental problems such as stress and anxiety in pregnant women. To achieve optimal effect, healthcare providers should consider content, session duration, and compliance when applying MBIs with pregnant women. Healthcare providers must master and teach intervention techniques successfully while concurrently monitoring and clarifying the impact of interventions on pregnant women.

### Conclusions

MBIs have differing effects on different mental health outcomes in pregnant mothers, including moderate effects on stress and anxiety and no effect on depression. In this review and meta-analysis, we found various MBIs are currently being applied to pregnant women that differ significantly in terms of technique, content, and session/program duration. MBIs may effectively improve the mental health of pregnant mothers and indirectly improve the health of mothers, their babies, and their families.
